# Understanding the mechanisms of silica nanoparticles for nanomedicine

**DOI:** 10.1002/wnan.1658

**Published:** 2020-06-29

**Authors:** Ziyuan Li, Yingwen Mu, Cheng Peng, Martin F. Lavin, Hua Shao, Zhongjun Du

**Affiliations:** ^1^ School of Medicine and Life Sciences University of Jinan‐Shandong Academy of Medical Sciences Jinan China; ^2^ Shandong Academy of Occupational Health and Occupational Medicine Shandong First Medical University & Shandong Academy of Medical Sciences Jinan China; ^3^ Queensland Alliance for Environmental Health Sciences (QAEHS) The University of Queensland Brisbane Queensland Australia; ^4^ University of Queensland Centre for Clinical Research (UQCCR) The University of Queensland Brisbane Queensland Australia

**Keywords:** biocompatibility, nanomedicine, silica nanoparticles

## Abstract

As a consequence of recent progression in biomedicine and nanotechnology, nanomedicine has emerged rapidly as a new discipline with extensive application of nanomaterials in biology, medicine, and pharmacology. Among the various nanomaterials, silica nanoparticles (SNPs) are particularly promising in nanomedicine applications due to their large specific surface area, adjustable pore size, facile surface modification, and excellent biocompatibility. This paper reviews the synthesis of SNPs and their recent usage in drug delivery, biomedical imaging, photodynamic and photothermal therapy, and other applications. In addition, the possible adverse effects of SNPs in nanomedicine applications are reviewed from reported in vitro and in vivo studies. Finally, the potential opportunities and challenges for the future use of SNPs are discussed.

This article is categorized under:

Nanotechnology Approaches to Biology > Nanoscale Systems in Biology
Therapeutic Approaches and Drug Discovery > Emerging Technologies

## Graphical abstract

Schematic diagram of silica nanoparticles applied to different nanomedicine.

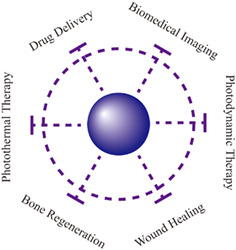

## 1 INTRODUCTION

Nanomedicine is the application of the principles and methods of nanoscience and technology to medicine, aimed at developing more sensitive and rapid medical methods and understanding the processes and mechanisms of life activities at the more micro or nano level (Albanese, Tang, & Chan, 2012; Bregoli et al., 2016; Emerich, 2005; Lloyd‐Parry, Downing, Aleisaei, Jones, & Coward, 2018). Worldwide it is evident that there is increasing emphasis on biomedical applications of a wide range of nanomaterials including silica nanoparticles (SNPs), superparamagnetic iron oxide nanoparticles, gold nanoparticles, and graphene and metal organic frameworks (Cheng, Deng, Lin, Cai, & Zink, 2019; Darweesh, Ayoub, & Nazzal, 2019; Rive et al., 2019; Yan et al., 2019; Zhong, Kankala, Wang, & Chen, 2019). Among nanomaterials, SNPs have great developmental potential and have become a research hotspot for their great developmental potential.

SNPs are inorganic engineered materials ranging in size from 1 to 100 nm. They have unique characteristics including large specific surface area, easy synthesis and amplification, facile surface modification, and robust delivery systems (Pasqua, Cundari, Ceresa, & Cavaletti, 2009; Patel et al., 2008; Qian & Bogner, 2012; Yamamoto & Kuroda, 2018; Yazdimamaghani, Barber, Hadipour Moghaddam, & Ghandehari, 2018). Compared to silicon nanoparticles (also known as silicon quantum dots), SNPs have gained more research attention on medicine applications (Joo et al., 2015; O'Farrell, Houlton, & Horrocks, 2006; Santos, Makila, Airaksinen, Bimbo, & Hirvonen, 2014). SNPs have been widely used in the field of nanomedicine and pharmacy, in particular in drug delivery system. Mesoporous silica nanoparticles (MSNs) have the ability to load a wide variety of fluorescent molecules while protecting dye molecules from harsh environmental conditions, thereby enhancing light stability and concentrating light signals with better resolution for diagnostic and monitoring purposes (Gimenez et al., 2015; Yang, Feng, & Liu, 2016). While SNPs are widely used, their safety has been of great concern for human health (Chen, Chen, & Shi, 2013). The main safety issue for the use of SNPs is their direct cytotoxicity, immune toxicity, and genotoxicity that affected particle size, potential aggregation, long‐term bioaccumulation, and hemolytic activity (Feng et al., 2019; Hadipour Moghaddam, Mohammadpour, & Ghandehari, 2019; Janssen et al., 2018; Paris, Baeza, & Vallet‐Regi, 2019). Furthermore, published data showed some inconsistencies between in vitro and in vivo studies (Murugadoss et al., 2017). To address this and other issues, this review provides a comprehensive overview of SNPs in nanomedicine. First, we briefly introduce the current preparation processes for SNPs followed by a focus on the functions and applications of SNPs in different fields of nanomedicine. For the safety concerns of SNPs, we review the data from nanotoxicology studies both in vivo and in vitro. Finally, we attempt to identify and summarize the opportunities and challenges for SNPs in other nanomedical applications. For other recent reviews (Jeelani, Mulay, Venkat, & Ramalingam, 2019; Narayan, Nayak, Raichur, & Garg, 2018; Wu, Mou, & Lin, 2013), we critically synthesize the current knowledge widely involved and review the opposing viewpoints from the perspective of in vitro and in vivo toxicology. At last, we give possible viewpoints in facing the potential unfavorable effects of the nanomaterials.

## 2 SYNTHESIS OF SNPs

### 2.1 Synthetic processes

#### 2.1.1 Stöber method

The Stöber method is one of the sol–gel methods, which was proposed by Stöber, Fink, and Bohn (1968). It has been widely used in the synthesis of SNPs since it can form monodisperse solid particles without a template (Liberman, Mendez, Trogler, & Kummel, 2014). The experimental method involves combining tetraethyl orthosilicate (TEOS) or other silicates with water, alcohol, and ammonia in a mixture to form a large number of silicic acid molecules. The silicic acid molecule is dehydrated or dealcoholized to form a Si–O–Si condensate. After the concentration reaches supersaturation, the primary SNPs rapidly aggregate to form primary silica particles which are further grown into more stable silica particles. Soluble silicic acid molecules or condensates continue to react and control the growth on its surface, and finally become SNPs (Blaaderen, Geest, & Vrij, 1992; Green et al., 2003).

Solid and mesoporous SNPs can be prepared by the Stöber method. SNPs are characterized by high uniformity and purity, excellent crystal shape, and optical transparency. However, the expensive raw materials required in the preparation process are expensive, time‐consuming and have the potential to cause environmental pollution. Therefore, more extensive studies with more economical raw materials and efficient production are required for the development of the Stöber method.

#### 2.1.2 Microemulsion method

Microemulsion is an isotropic, surfactant stable, and thermodynamically stable system composed of two incompatible liquids, and is considered as a synthetic “nanoreactor” (Wolf & Feldmann, 2016). Reactants usually form a microemulsion as an aqueous phase and then contact with another reactant to form nanoparticles (NPs) (Boutonnet, Kizling, Stenius, & Maire, 1982). The particle shape of the NPs prepared by this method is narrow, and different particle sizes can be obtained by changing the different parameters of the microemulsion system.

SNPs with a particle size below 100 nm can be prepared by the microemulsion method. The experimental device is simple and easy to operate and the particle size distribution and shape of the SNPs can be controlled by changing the experimental conditions. The method could be improved if a higher purity of SNPs could be obtained by removing the organic components produced during the synthesis process (Aubert et al., 2010).

#### 2.1.3 Gas phase method

The gas phase method generally uses silicon tetrachloride (tetrachlorosilane) as a raw material, and hydrolyzes silicon tetrachloride gas with hydrogen and oxygen at a high temperature to obtain a fumed silica, and uses a sol atomizer to obtain a polydisperse or monodisperse liquid (Lu et al., 1999). The droplets are further heat‐treated to obtain the desired NPs with a low density and unique mesoporous structure. The advantages of preparing SNPs by the gas phase method are high purity, high dispersion, and less hydroxyl groups on the surface. However, the disadvantages are expensive raw materials, high energy consumption, complex technology, and specialized equipment (Buesser & Pratsinis, 2012). Because of their excellent reinforcing properties, the NPs produced by this method are mainly used to enhance the rubber properties (Cohen‐Addad, Roby, & Sauviat, 1985).

#### 2.1.4 Precipitation method

The precipitation method involves different chemical components in the solution state and subsequently adding the precursor precipitant to the mixed solution to form the precipitate (Nele, Vidal, Bhering, Carlos Pinto, & Salim, 1999). The precipitate is then dried or calcined into SNPs. The production process involves simple steps and less consumption of energy, but impurities and aggregation can readily occur. Therefore, an organic dispersion is required in the silicate solution, which may lead to good dispersion of the SNPs.

Comparison of the synthesis processes is listed in Table 1.

**TABLE 1:** Comparison of the four synthesis processes

Synthetic processes	Schematic diagram	Characteristic
Stöber method	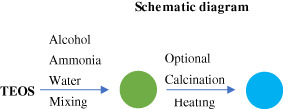	Particle uniformity; high purity; good optical transparency
Microemulsion method	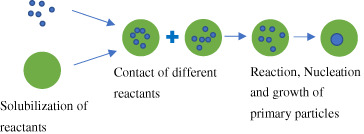	Particle size below 100 nm; simple operation; homogeneous inorganic compound powder
Gas phase method	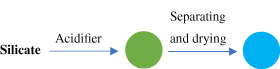	High porosity; low thermal conductivity; high cost
Precipitation method	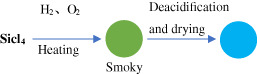	Solid particles; large particle size; suitable for industrial production

### 2.2 Mesoporous silica nanoparticles

**FIGURE 1:**
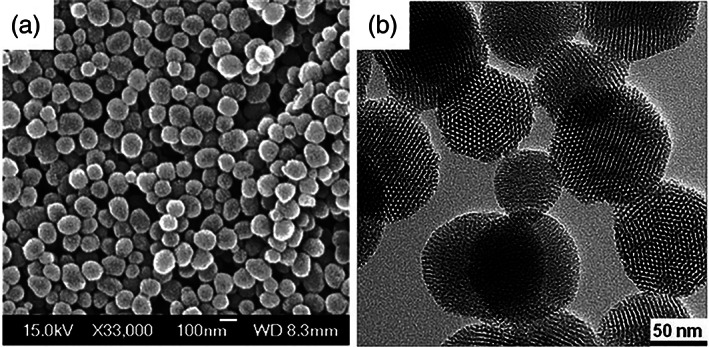
(a) SEM image of MCM‐41 mesoporous silica nanoparticles; (b) TEM image of MCM‐41 (Reprinted with permission from Li et al. (2012). Copyright 2012 Royal Society of Chemistry)

MSNs are widely used in nanomedicine because of their high porosity and biocompatibility, easy surface modification, and self‐adjuvanticity (Nguyen, Choi, & Kim, 2019). Methods for preparing MSNs with good dispersion properties used in medicine include fast self‐assembly, soft and hard templating, a modified Stöber method, dissolving reconstruction, and modified gas phase method (Wu et al., 2013). The most commonly used method is the Stöber method (L. Chen, Zhou, & He, 2019). As an example, Grün, Lauer, and Unger (1997) used an improved Stöber synthesis to prepare submicron MCM‐41 particles. Subsequent to that, 100 nm MCM‐41 SNPs were obtained by using a diluted surfactant solution (Cai et al., 2001). MSNs less than 50 nm were obtained by adding a hydrochloric acid solution to the previous cetyl trimethylammonium chloride and a triblock copolymer as cationic and nonionic surfactants to the typical Stöber method (Li, Barnes, Bosoy, Stoddart, & Zink, 2012; Suzuki, Ikari, & Imai, 2004). Among the various MSNs, MCM‐41 particles are widely studied in nanomedicine (Figure 1). MCM‐41 particles are synthesized by using TEOS or sodium metasilicate as a silica precursor, the surfactant cetyltrimethylammonium bromide (CTAB) as a liquid crystal template and an alkali as the catalyst (Li et al., 2012; Tang, Li, & Chen, 2012).

**FIGURE 2:**
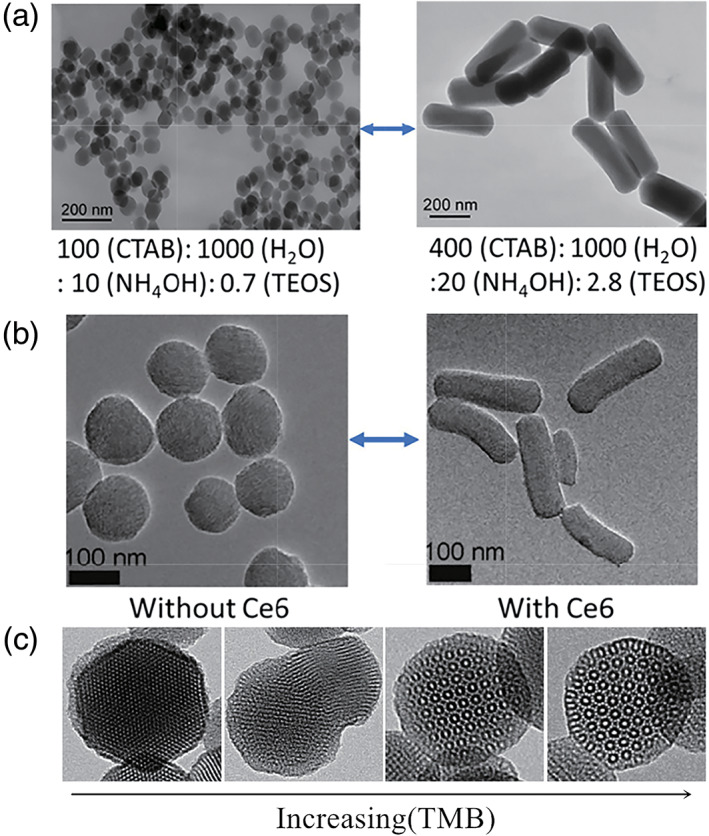
TEM images: (a) molar reagent ratio, (b) the dopant to silica networks, (c) TEM images of MSNs prepared from TEOS/AEAPTMS with 12, 29,47, and 116 mM mesitylene (TMB), using a constant ammonium hydroxide concentration (13.8 mM) and stirring rate (650 rpm) (Reprinted with permission from Huang, Teng, Chen, Tang, and He (2010). Copyright 2010 Springer; Sun et al. (2017). Copyright 2017. Nature Publishing Group; Yang, Gong, Qian, et al. (2015). Copyright 2015 Elsevier)

These reaction parameters can be freely adjusted to produce stable and monodisperse MSNs with various shapes, pore structures, and particle sizes. The shape of MSNs can be directly controlled by changing the morphology of the CTAB template (Slowing, Trewyn, Giri, & Lin, 2007). Rod‐shaped CTAB micelles can be obtained by carefully selecting the reagent composition ratio of the CTAB, water, and pH of the solution (using ammonia or sodium hydroxide) (Slowing, Vivero‐Escoto, Trewyn, & Lin, 2010; Yu, Greish, McGill, Ray, & Ghandehari, 2012). Similarly, doping a photosensitizing molecule chlorin e6 (Ce6) into MSNs during the sol–gel process shows that the morphology of the mesoporous silica changes from spherical to rod‐shaped (Yang, Gong, Qian, et al., 2015). The rod‐shaped particles are shown in Figure 2a,b. Previous studies have reported 12‐corner SNPs (Sun et al., 2017). The CTAB micelle size and size distribution were systematically altered by adjusting the concentration of the micelle pore expander, mesitylene (TMB). The Transmission Electron Microscope (TEM) image shows that as the TMB concentration increases, the MSN structure changes from a hexagon to a multichamber to a cube, and finally becomes a 12‐corner symmetrical structure (Figure 2c).

### 2.3 Core–shell SNPs

Another research group developed a technology that is most commonly used for the synthesis of core–shell SNPs (Yang, Lind, & Trogler, 2008). They first coated polystyrene templates with poly‐l‐lysine and then performed a polycondensation reaction with hydrolyzed tetramethyl orthosilicate. After calcination, the hollow nanoshells had a 6–10 nm thick shell and the diameter of the NPs isotropically decreased 10–20%. The core could be composed of metals for templating, which produces core‐shell SNPs (Vivero‐Escoto, Huxford‐Phillips, & Lin, 2012). Wang, Tang, Zhao, Wan, and Chen (2014) prepared the magnetite–silica core–shell nanoparticles (Fe_3_O_4_@SiO_2_NPs) by direct oxidation from silicon powder. This technology provides a new opportunity for the preparation of core shell NPs for biomedical applications. Herein, Reis et al. (2019) develop a new polymeric coating for the gold–core SNPs by combining different ratios (25/75, 50/50, and 75/25) of two materials, poly‐2‐ethyl‐2‐oxazoline and β‐cyclodextrin (β‐CD). This improved the biological properties of the gold–core silica shell NPs and provided new ideas for cancer treatment.

## 3 NANOMEDICINE APPLICATIONS

### 3.1 Drug delivery

**FIGURE 3:**
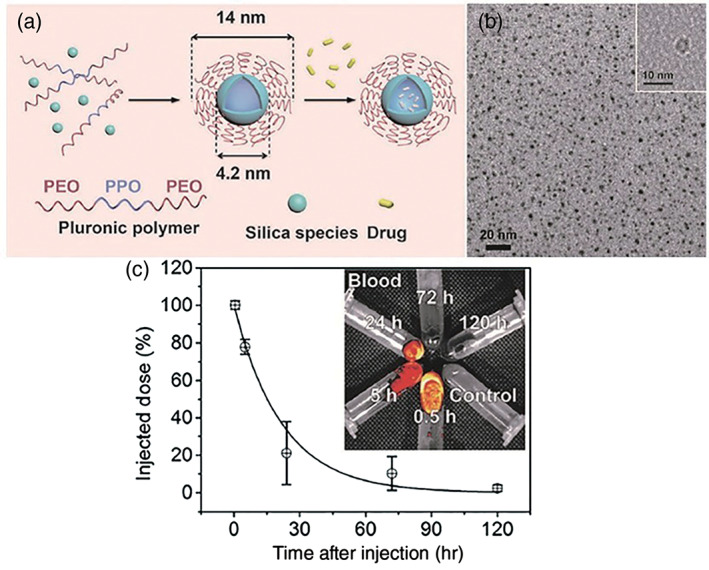
Schematic illustration (a) and TEM images of silica cross‐linked F108 micelles (b). Blood‐circulation time of silica cross‐linked F108 micelles determined by ICP‐OES (c) (Reprinted with permission from Niu et al. (2017). Copyright 2017 Royal Society of Chemistry; Zhao et al. (2016). Copyright 2016 Wiley)

**FIGURE 4:**
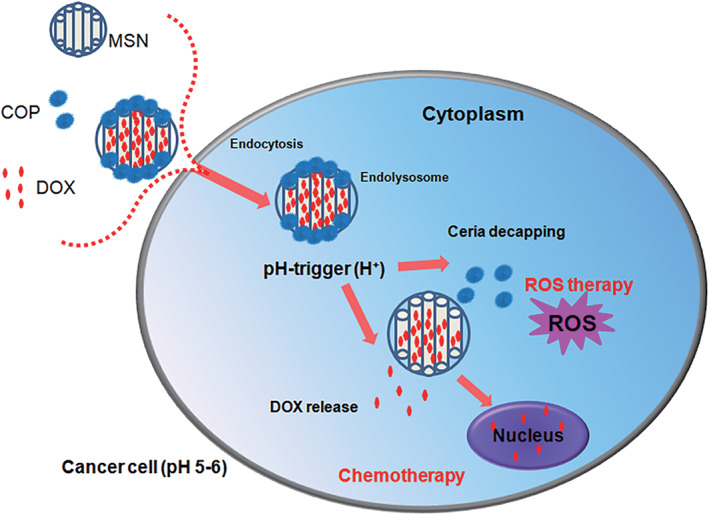
Schematic illustration shows the intracellular ROS therapy and chemotherapy of DOX‐loaded COP@MSNs system triggered by pH in cancer cells (Reprinted with permission from Singh et al. (2019). Copyright 2019 ACS Publications)

Since 2001 when MSNs were first proposed as potential drug delivery systems (Vallet‐Regi, Rámila, del Real, & Pérez‐Pariente, 2001), different SNPs have been widely used in drug delivery (Vallet‐Regi & Tamanoi, 2018). Table 2 summarizes several SNPs‐based drug delivery systems, including instant drug delivery systems, sustained drug delivery systems, stimulus response control drug delivery systems, and targeted drug delivery systems. Additional studies successfully synthesized a controlled release system composed of MSNs with covalently bound dipalmitoyl moieties supporting phosphorylated lipids. The system could release a model molecule, fluorescein from the pores of lipid bilayer coated MSNs (LB‐MSNs) via a chemically triggered disulfide reduction (Roggers, Lin, & Trewyn, 2012). The system indicated that LB‐MSNs could potentially be used to design a drug delivery system capable for controlled release. In 2013, this group further developed a drug delivery system for photosensitive cooperative treatment of cancer with cytotoxic cadmium sulfide nanoparticles and camptothecin (Knezevic & Lin, 2013). The system was further optimized by Zhao et al. (2016) who reported the Cy‐5.5‐labeled silica cross‐linked PEO132‐PPO50‐PEO132 (F108) micelles with 14 nm ultra‐small fluid dynamic particle size. These micelles provide a stronger drug diffusion barrier from the polymer core across the silica cross‐linking layer, exhibiting a half‐life in the blood circulation for up to 19 hr. This lead to efficient accumulation through the well‐known enhanced permeability and retention effect in subcutaneously transplanted tumor models (Niu, Li, & Shi, 2017) described in Figure 3. Li, Zhang, Sheng, and Sun (2017) proposed SNPs with a thyroid stimulating hormone receptor targeting specific targets for thyroid cancer. Doxorubicin (DOX) nanoparticles can be triggered by an acid to release the drug payload for cancer treatment. Hadipour Moghaddam, Yazdimamaghani, and Ghandehari (2018) prepared glutathione (GSH)‐sensitive hollow mesoporous NPs (HMSiO_2_ NPs) using a selective etching strategy based on structural differences. These particles were compared with non‐GSH‐sensitive TEOS HMSiO_2_ in terms of synthesis methods, characterization, DOX release curves, and in vitro cytotoxicity to McF‐7 breast cancer cells. These results showed that HMSiO_2_ NPs had the advantages of high load capacity and controlled degradation at 100 μg/ml concentration and the particles can hold 9 μg/ml of DOX and effectively kill cancer cells. Juthani et al. (2020) functionalized ultrasmall fluorescent core–shell SNPs, referred to as Cornell prime dots (C′dots), with v integrin‐binding (cRGDY), or nontargeting (cRADY) peptides, and PET labels (^124^I, ^89^Zr) and investigated the utility of dual‐modality cRGD‐C′ dots for enhancing accumulation, distribution, and retention (ADR) in a genetically engineered mouse model of glioblastoma (mGBM). As a result, there was an improvement in brain tumor drug delivery and penetration, and an enhancement of ADR were achieved. Singh et al. (2019) prepared MSNs decorated with cerium oxide nanoparticles (COP@MSNs) using a pH‐triggered response mechanism to control drug release and intracellular drug delivery. They blocked the mesopores of the carboxyl‐functionalized MSNs with aminated COP. These pores were opened under acidic conditions to release the loaded drug and to establish a pH responsive drug delivery system (Figure 4). With the unique properties of SNPs and trigger response mechanisms (pH sensitive, light sensitive, magnetically sensitive, etc.), SNPs play a potential role in the treatment of diseases, especially in the treatment of tumors.

**TABLE 2:** Comparison of the SNPs‐based drug delivery systems

Drug delivery systems	Type	Characteristic	Model drug	References
Immediate drug delivery systems (IDDSs)		Improving dissolution rate of hydrophobic drugs	Telmisartan (TEL), prednisolone, etc.	Zhang et al. (2012), Sen Karaman et al. (2018)
Sustained drug delivery systems (SDDSs)	Unmodified; modified	Providing long‐term drug release	Felodipine, nonsteroidal anti‐inflammatory, etc.	Zhang et al. (2012), Ahmadi, Dehghannejad, Hashemikia, Ghasemnejad, and Tabebordbar (2014)
Stimuli‐responsive controlled drug delivery systems (CDDSs)	*Endogenous*: pH, redox, enzyme, glucose, H_2_O_2_, ATP *Exogenous*: Thermo, light, magnetic, ultrasound, electro	Effective control of drug release	β‐Cyclodextrin, dexamethasone, etc.	Meng et al. (2010), Giri, Trewyn, Stellmaker, and Lin (2005), Deng et al. (2011)
Targeted drug delivery systems (TDDSs)		Targeted delivery of drugs, less dosage, tumor drug	Doxorubicin, camptothecin, TNF‐α protein, etc.	Ashley et al. (2011), Porta et al. (2013)

### 3.2 Biomedical imaging

Compared to traditional materials, SNPs exhibit enhanced images in biomedical imaging characteristics (Cho, Glaus, Chen, Welch, & Xia, 2010; Choi & Frangioni, 2010). In recent years, studies on the application of SNPs to imaging techniques have been reported (Vivero‐Escoto et al., 2012). These studies indicated that SNPs are powerful in making contrast agents of the chemical stability under various physiological conditions, high sensitivity and selectivity for the target, and good image contrast. In addition, having a long delay, SNPs have sufficient circulation time in blood. Tumor imaging is an important part of biomedical imaging. Another study found that nanoparticles administered by intravenous injection gave a strong fluorescence in tumors and magnetic resonance imaging (MRI) and computed tomography (CT) signals were enhanced (Yang, Gong, Liu, et al., 2015). Wu et al. (2008) were first to report the use of MSNs for bimodal imaging in animal models. Chen et al. (2014) combined copper‐64 and 800 CW onto MSNs, and successfully imaged tumor blood vessels in vivo using positron emission tomography (PET) imaging and near‐infrared imaging techniques using a multimode approach. The results showed that both in vivo PET and fluorescence imaging resolutions are significantly improved with this combination. In addition, fluorescently labeled mesoporous silica nanoparticles are also used as an endoscopic contrast agent for the diagnosis of precancerous lesions of the colon.

**FIGURE 5:**
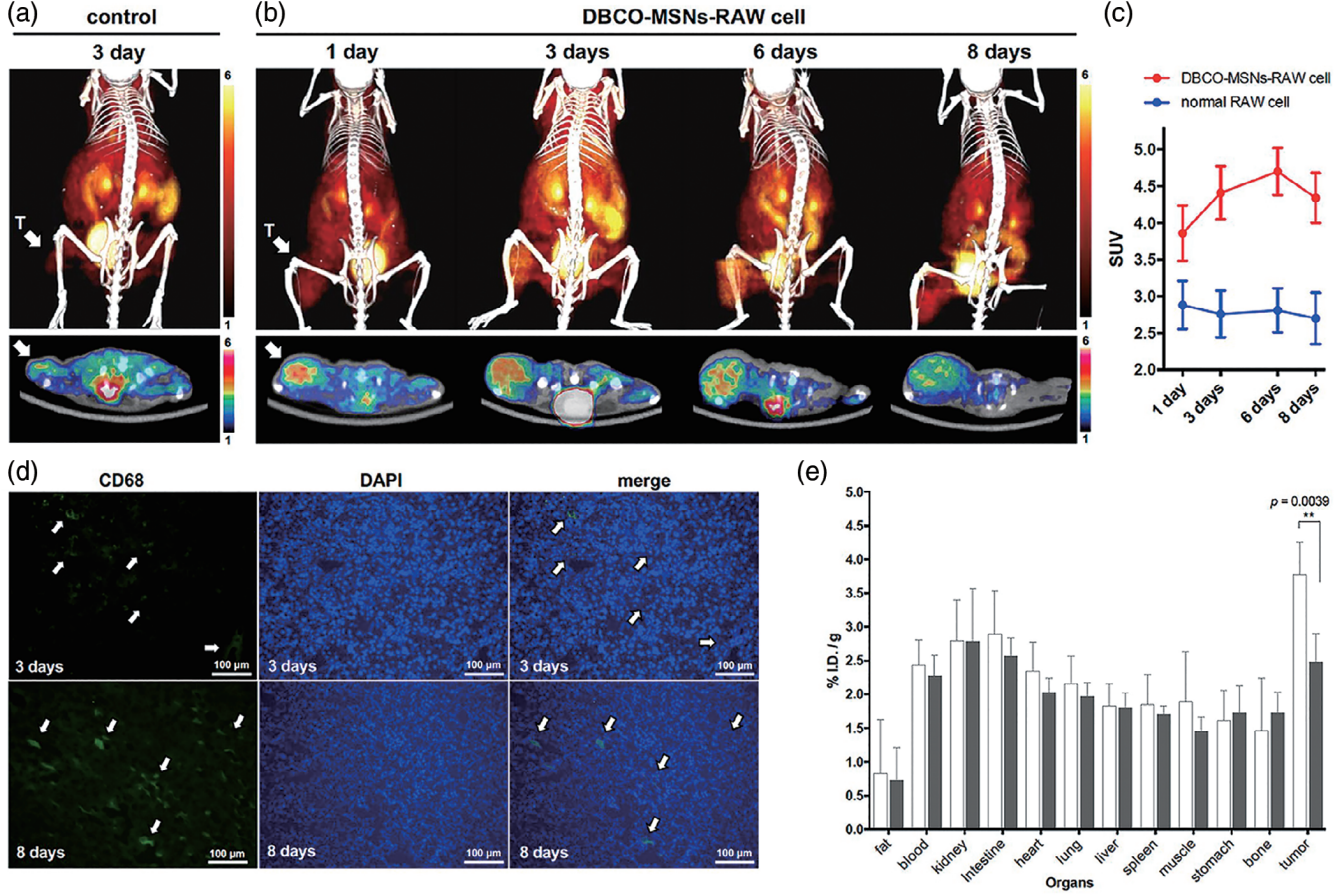
Macrophage cell tracking PET imaging via bioorthogonal^18^F‐labeling in a tumor model. (a,b) Three‐dimensional reconstruction (upper) and transverse section (lower) combined PET‐CT images of 18F‐labeled azide ([^18^F]2; 11.1 MBq) in U87 MG tumor‐bearing mice treated with only normal RAW 264.7 cells (2 × 10^6^ cells) 3 days earlier (control study; a) or in mice treated with DBCO‐MSNs‐RAW cells (2 × 10^6^ cells; 0.1 ng Si/cell) 1, 3, 6, or 8 days earlier (cell tracking study; b), recorded 1 hr after injection of [^18^F]2. T = tumor, and (c) standardized uptake values (SUVs) in tumor area. (d) Immunofluorescence analysis of RAW264.7 cells in tumor tissue obtained from U87 MG tumor‐bearing mice treated with DBCO‐MSNs‐RAW cells 3 and 8 days earlier. (e) Biodistribution of [^18^F]2 inU87MG tumor‐bearing mice treated with DBCO‐MSNs‐RAW cells (cell tracking; white bar) or only RAW 264.7 cells 3 days earlier (control; gray bar), measured 1 hr after injection (Reprinted with permission from Jeong et al. (2019). Copyright 2019 Elsevier)

Khatik et al. (2019) reported MRI for tissue‐specific T1/T2 double contrast. They developed a fancy kind of magnus nano‐bullets (Mn‐DTPA‐F‐MSNs) distinguished by a magnetic (Fe_3_O_4_‐NPs) head combined with mesoporous silica (SiO_2_) that persisted in the body. Subsequently, they modified the mesoporous SiO_2_ group and finally loaded them with Mn^2+^. The results show that the magnus nano‐bomb enhances the T1‐weighted MRI effect of redox reaction in GSH in vitro and in vivo. Jeong et al. (2019) designed a PET imaging protocol that promoted an in vivo strain‐promoted alkyne azide cycloaddition (SPAAC) covalent labeling reaction in macrophages in vivo to track macrophages by using aza‐dibenzocyclooctyne‐tethered PEGylated mesoporous silica nanoparticles (DBCO‐MSNs) with the short half‐life F‐18‐labeled azide‐radiotracer. This imaging scheme forms ^18^F‐labeled azadibenzocycloocta‐triazolic MSNs (^18^F‐DBCOT‐MSNs), in RAW 264.7 cells by an orthogonal SPAAC reaction of DBCO‐MSNs with [^18^F] fluoropentaethylene glycolic azide ([^18^F]2). Thus, the macrophages successfully migrated to the tumor site. Finally, the results of tissue radioactivity distribution were consistent with those of PET imaging (Figure 5). Besides, a new cup‐shaped SNP was found to be useful for ultrasonic imaging (F. Chen, Ma, et al., 2017). The novel cup‐shaped SNP also called an exosome‐like silica nanoparticles (ELS) was prepared by emulsion templating. They compared ELS with other SNPs and found that ELS strengthened particle echogenicity as well as improved the cell labeling efficiency. It also increased the in vitro and in vivo echogenicity of stem cells, providing clues to the use of further application of SNPs in ultrasound imaging. In addition, their team reported an ultrasound‐capable biodegradable SNPs that showed no cytotoxicity at the 250 μg/ml concentration required for labeling, and cleared from cells in approximately 3 weeks (Kempen et al., 2015).

Table 3 shows the difference among optical imaging, MRI, PET, CT, and ultrasound imaging. These five bioimaging technologies based on SNPs have both advantages and disadvantages. For example, the image resolution of a simple and fast optical imaging is low and CT imaging with high spatial resolution may have a radiation risk. However, the combination of multimodal imaging techniques may improve individual imaging techniques. A study showed that mesoporous silica‐based triple‐modal nanoprobes could be successfully applied to long‐term imaging of tumor draining sentinel lymph nodes (Huang et al., 2012). The imaging results of different modalities are consistent and complementary because of the high stability and robustness of nanoprobes. In addition, biocompatibility is an important condition for the application of SNPs‐based developers in nanomedicine in any imaging technologies. Park et al. (2010) not only successfully used MRI and optical imaging to identify mesenchymal stem cells (MSCs) labeled with multimodal imaging nanoprobes, but also proved the biocompatibility of nanoprobes.

**TABLE 3:** Comparison of the different bioimaging technology

Bio‐imaging	Imaging medium	Advantages	Disadvantages	Contrast medium (SNPs)	References
Optical imaging	Fluorescence	High sensitivity, multicolor imaging, simple operation, low cost	Low spatial resolution, poor tissue penetration	Fluorescent dye‐conjugated MSNs, CDs@MSNs, etc.	Heidegger et al. (2016), Fu et al. (2015)
Magnetic resonance imaging (MRI)	Magnetic field (radio waves)	High spatial resolution, no tissue penetrating limit, harmless	High cost, low sensitivity, long imaging time, hard calculating	Fe_3_O_4_@MSNs, HMnO@MSNs, MSN‐DTTA‐Gd, etc.	Kim et al. (2008), Kim et al. (2011), Taylor et al. (2008)
Positron emission tomography (PET)	γ‐ray	High sensitivity, quantitative, no tissue penetrating limit, whole‐body‐scanning	Radiation risk, low spatial resolution	DBCO‐PEG‐MSNs, ^89^Zr‐MSNs, etc.	Lee, Kim, Jeong, et al. (2013), F. Chen, Goel, et al. (2015)
Computed tomography (CT)	X‐ray	High spatial resolution, no tissue penetrating limit	Radiation risk, unquantifiable analysis	Au@MSNs, FePt@MSN, etc.	Song et al. (2015), F. Chen, Ma, et al. (2017)
Ultrasound imaging	Ultrasonic waves	Real‐time, low cost	Multiple influencing factors, low resolution	MSN‐PFH, ELS, etc.	Wang et al. (2012), F. Chen, Ma, et al. (2017)

### 3.3 Photodynamic therapy

**FIGURE 6:**
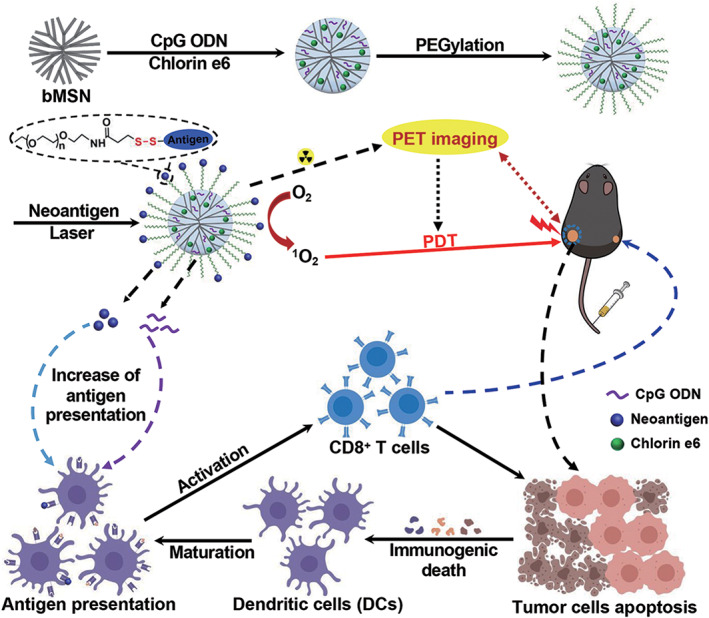
Schematic illustration of fabrication of bMSN (CpG/Ce6)‐neoantigen and mechanism of bMSN (CpG/Ce6)‐neoantigen nano‐vaccines for PDT‐enhanced cancer immunotherapy (Reprinted with permission from Xu et al. (2019). Copyright 2019 ACS Publications)

Photodynamic therapy (PDT) is an effective, noninvasive treatment for local tumors that are accessible to light sources. PDT activates light‐sensitive molecules (photosensitizers) by lasers of a specific wavelength and then produces cytotoxic reactive oxygen species (ROS), which ultimately leads to vascular closure and tumor cell death (Abrahamse & Hamblin, 2016; Lucky, Soo, & Zhang, 2015; Wang, Tao, Cheng, & Liu, 2011). PDT usually requires synergy with other biomedical methods to achieve optimal results. To achieve this, W. H. Chen, Luo, et al. (2017) constructed a multifunctional nanoplatform (TPZ@MCMSN‐Gd^3+^) used in in vivo studies to demonstrate that under the guidance of near‐infrared fluorescence/MRI, TPZ@MCMSN‐Gd^3+^ exhibited preferential aggregation at the tumor site through the synergistic action of PDT and bio‐induced chemotherapy, leading to significant inhibition of tumor progression. Yang et al. (2017) adsorbed the photosensitizer chloramphenicol e6 (Ce6) and the antitumor drug (DOX) on magnetic mesoporous silica nanoparticles (M‐MSNs) and showed nanocomposites could enhance the ability of MRI and CT imaging to allow for real‐time imaging to guide the combination of PDT and chemotherapy to achieve a highly effective synergistic anti‐tumor effects in vivo. Tumor‐bearing Balb/c mice were used as an animal model for PDT and chemotherapy, and achieved synergistic anti‐tumor effects in vivo. Hu et al. (2018) developed a new self‐contained oxygen PDT platform‐zeolite‐catalase‐MB nanocapsule (ZCM nanocapsule) that accurately implanted ZCM nanocapsules into the tumor area under real‐time ultrasound imaging guidance and near‐infrared laser irradiation, resulting in complete killing of local pancreatic cancer (PC) cells. In addition, Zhang et al. (2016) reported that combined synergistic treatment can effectively evade drug resistance. However, the use of PDT to treat disseminated, metastatic cancer remains challenging. Xu, Nam, Hong, Xu, and Moon (2019) developed a multifunctional nanomaterial system using a combination of PDT and individualized cancer immunotherapy, which provides new hope for the resolution of systemic disseminated and metastatic tumors (Figure 6).

### 3.4 Photothermal therapy

**FIGURE 7:**
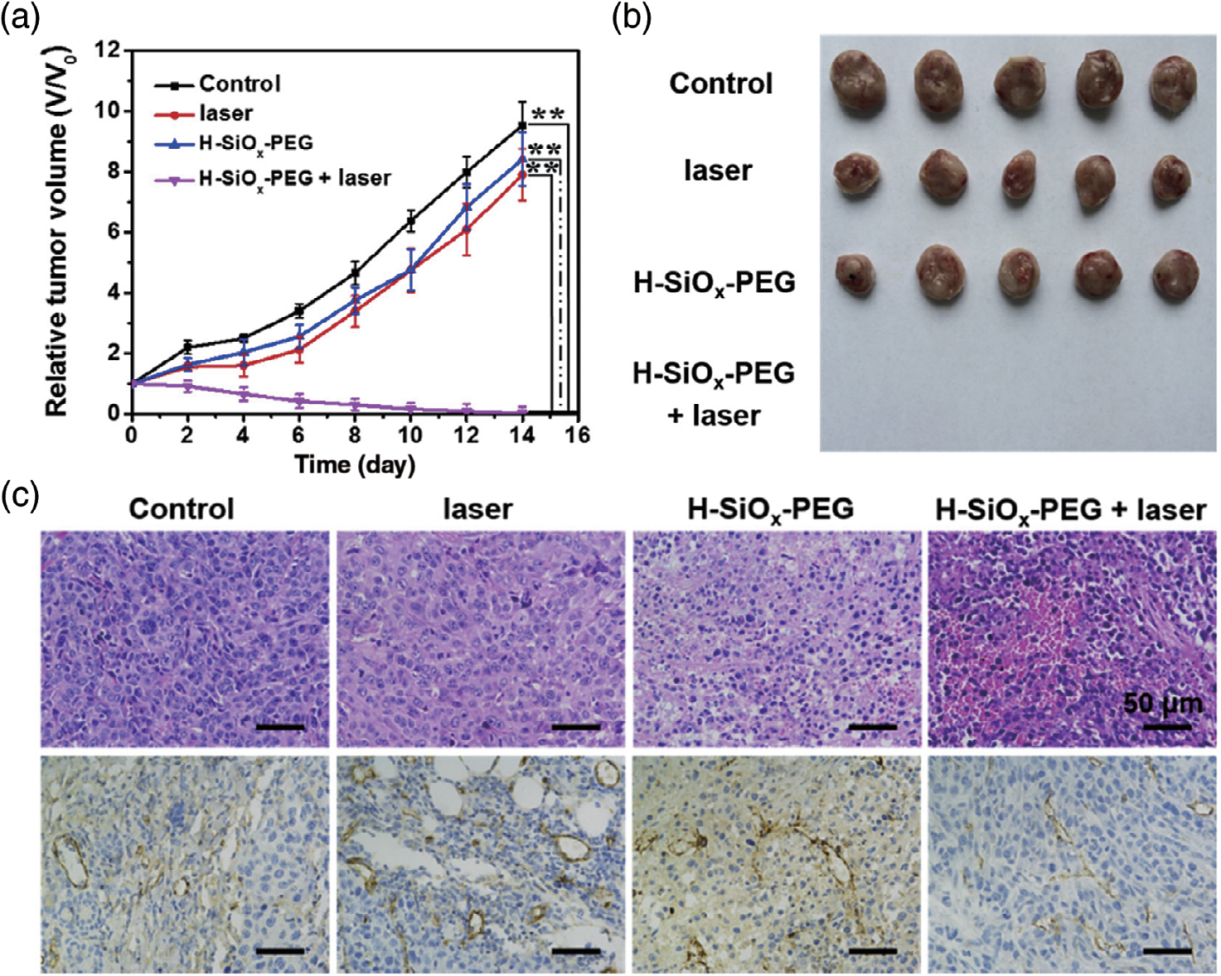
(a) Tumor growth curves of mice with different treatments. (b) Representative photographs of tumors obtained from mice at day 14 after different treatments. (c) H&E staining (top row) and CD31 staining (bottom row) of tumor slices at day 2 in mice of each group after treatment (Reprinted with permission from Yu et al. (2017). Copyright 2017 Elsevier)

Photothermal therapy (PTT), a method which uses light‐absorbing nanomaterials to convert radiation into heat for tumor ablation, is an ideal adjuvant therapy in the treatment of tumors because of its controllability, simplicity and non‐invasiveness (Menon et al., 2013; Wang et al., 2020). Yu, Yang, Chen, and Li (2017) reported that synthetic non‐stoichiometric hollow silica nanoparticles (H‐SiO_*x*_ NPs) produce high photothermal conversion efficiency under 1,064 nm laser irradiation, which is highly effective for cancer treatment in vivo (Figure 7). Wang et al. (2019) prepared a multifunctional Janus structure of gold triangle‐mesoporous silica nanoparticles (NPs) as a multifunctional platform to deliver a hypoxia‐activated prodrug (TPZ) for topical PTT. In this method coupling of folate‐linked polyethylene glycol provides the Janus nanoplatform with targeting and minimal operational characteristics for the treatment of liver cancer. in vitro and in vivo experiments demonstrated that combined radiation sensitivity and photothermal anti‐tumor effects of the Janus nanoplatform, provide an effective and safe strategy for the treatment of liver cancer (Zhu et al., 2017).

However, PTT is ineffective for the ablation of certain tumor cells and can cause damage to healthy tissues (Li, Mivechi, & Weitzel, 1995), which may be due to an intracellular “autophagy” mechanism (White, 2015). In order to resolve this problem, T. Chen, Cen, et al. (2019) investigated bismuth crystal‐embedded silica (Bi@SiO_2_) nanoparticles, loaded with an autophagy inhibitor (chloroquine, CQ). They demonstrated that CQ molecules are transported intracellularly using SNPs, which significantly attenuated the degradation of autolysosomes by lysosomes in tumor cells, thereby inducing an inhibition of autophagy and resistance to PTT. These studies compensated for the shortcomings of PTT, and the combined effects achieved under milder near‐infrared radiation conditions have greatly improved both in vitro and in vivo anti‐tumor effects.

### 3.5 Others applications

**FIGURE 8:**
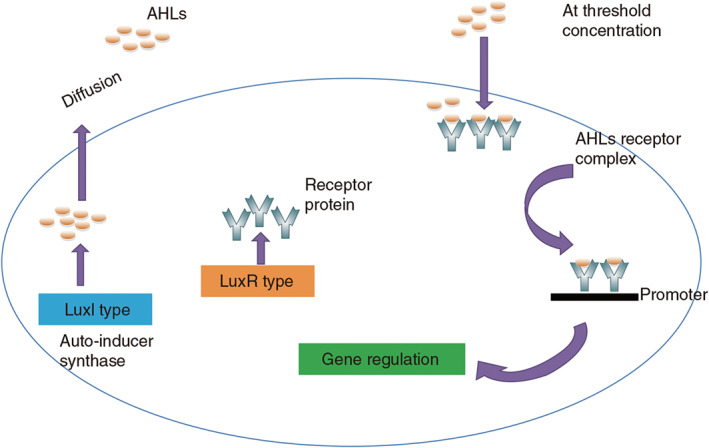
Quorum sensing in Gram‐negative bacteria. LuxI protein produces a signaling molecule termed as acyl homoserine lactones (AHL). With increasing the population density, an increase in the concentration of AHL is also observed. When it reaches to a certain threshold level, AHLs enter the cell and subsequently bind to LuxR‐type proteins to activate the expression of target genes (Reprinted with permission from Hayat et al. (2019). Copyright 2019 Future Science Group)

In addition to the main applications outlined above, SNPs also play an effective role in anti‐bacterial infection, wound healing, and the promotion of bone regeneration (Jia et al., 2019; Nethi, Das, Patra, & Mukherjee, 2019; Zhao et al., 2019). In the application of anti‐bacterial infection, SNPs can combine the characteristics of large surface area/pore volume, adjustable pore size and capacity for controlling release, and better control for the loading and release of antibacterial agents, to improve the effect of anti‐bacterial infections (Fernandez‐Moure, Evangelopoulos, Colvill, Van Eps, & Tasciotti, 2017). Gonzalez et al. (2018) treated bacterial infections with nanocarriers containing antibiotics that could penetrate bacterial walls. They combined polycationic dendrimers with levofloxacin‐loaded MSNs to trigger effective antibacterial action against Gram‐negative bacterial biofilms. When SNPs loaded antimicrobial peptides such as ll‐37 (Izquierdo‐Barba et al., 2009), non‐steroidal anti‐inflammatory drugs (Scavo et al., 2015), and antibiotics (Perelman, Pacholski, Li, VanNieuwenhze, & Sailor, 2008), were placed in the center of a bacteria‐containing AGAR gel, neither Gram‐positive *Staphylococcus aureus* nor Gram‐negative *Escherichia coli* showed obvious signs of bacterial growth. In another study, the quorum quenching potential of metal and metal oxide NPs were employed using targeted QS regulated virulence of Gram‐negative bacteria (Hayat et al., 2019). Figure 8 is a schematic diagram of quorum sensing in Gram‐negative bacteria. Miller (2015) prepared functionalized SNPs carrying the quenching molecule (β‐CD) and the results showed that the functionalized SNPs of cyclodextrin could persist in the bacterial cell environment and quenched the extracellular bacterial communication molecules, finally achieving the antibacterial effect.

**FIGURE 9:**
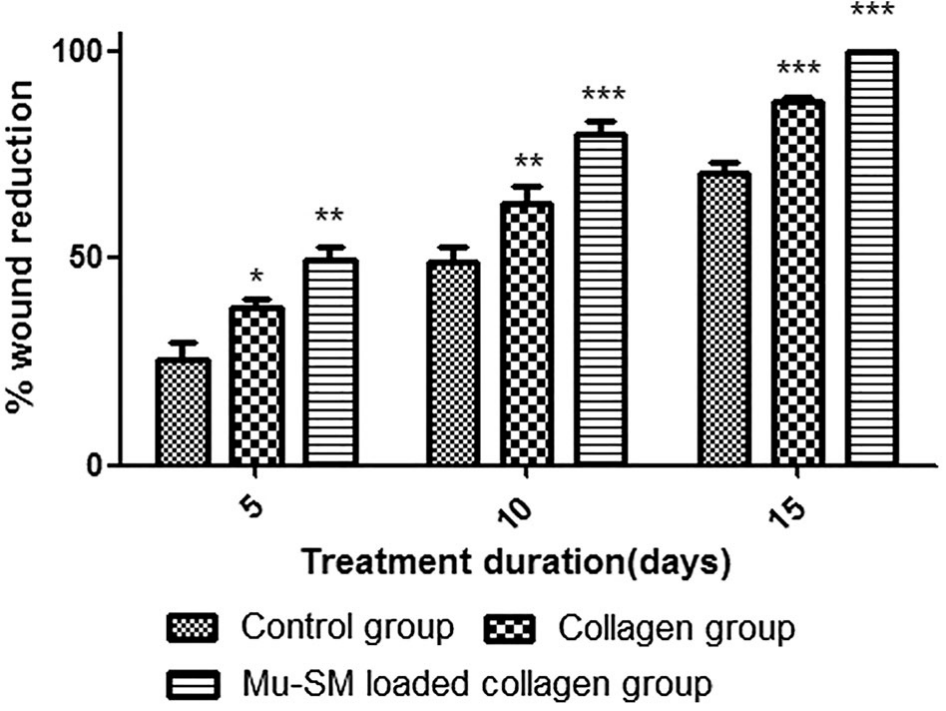
Percentage wound contraction for control (untreated wound), collagen and Mu‐SM collagen treatment. Significant difference in the efficacy was observed throughout the treatment duration for both collagen and Mu‐SM loaded collagen compared to the untreated group (Reprinted with permission from Perumal et al. (2014). Copyright 2014 Elsevier)

In relation to local application for wound healing, Quignard, Coradin, Powell, and Jugdaohsingh (2017) reported the effect of SNPs on proliferation and migration of human skin fibroblasts (CCD‐25SK). The authors found that SNPs were absorbed by fibroblasts and dissolved inside the cells, releasing silicic acid, which ultimately promotes wound healing (Nethi et al., 2019). Perumal, Ramadass, and Madhan (2014) combined three materials, collagen, mupirocin, and sol–gel treated silica microspheres in a coordinated way to establish a wound healing material as a scaffold. The results showed that Wistar rats with biomedical composite had a shorter time to complete wound epithelialization than the control group (Figure 9). Furthermore, the composite exhibits good water absorption, sustained drug availability, and antibacterial activity. Therefore, SNPs biomedical composite may be a promising strategy for wound healing in the future.

**FIGURE 10:**
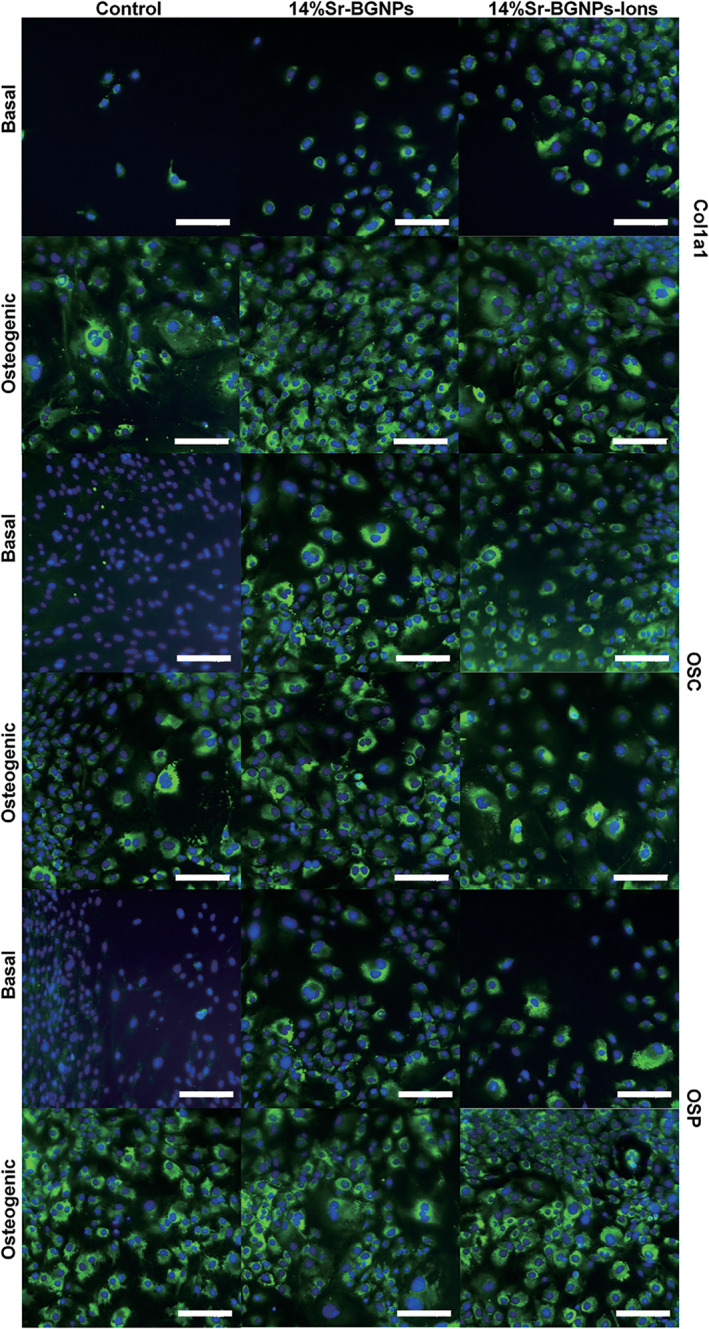
Fluorescence image, DAPI (blue), Col1a1, OSC, and OSP staining (all green) of MC3T3‐E1 cells exposure to 14% Sr‐BGNPs and their ionic release products (Sr‐BGNP concentration at 250 μg/ml) in basal and osteogenic conditions (3‐week culture period). Scale bar 150 mm (Reprinted with permission from Naruphontjirakul et al. (2018). Copyright 2018 Elsevier)

In addition, SNPs plays an important role in bone regeneration. Beck Jr. et al. (2012) found that SNPs mediated an inhibitory effect on osteoclasts and a stimulating effect on osteoblasts in vitro. SNPs significantly increased bone mineral density in mice. Weitzmann et al. (2015) also reported that the 50 nm SNPs could regulate the differentiation of osteoblasts and inhibit the differentiation of osteoclasts. in vivo models of mice, bone density, bone volume, and biochemical markers of bone formation were significantly increased. These results indicate that SNPs have certain biological activity on bone regeneration. In recent studies, Jia et al. (2019) synthesized mesoporous silica coated magnetic (Fe_3_O_4_) nanoparticles (M‐MSNs) and found that they had the ability to promote osteogenic differentiation and bone regeneration with bone marrow MSCs in a rat distraction osteogenesis (DO) model. Mora‐Raimundo, Lozano, Manzano, and Vallet‐Regi (2019) developed a system based on MSNs coated with poly(ethylenimine). This system was capable of transporting SOST siRNA and osteogenic peptide into the cell, and SOST siRNA silenced the osteoporosis related gene SOST (SOST gene inhibits Wnt signaling pathway and reduces osteoblastic differentiation), so as to promote bone formation. Another group of researchers, prepared SNPs (90 ± 10 nm) and added calcium and strontium to produce monodispersed strontium containing bioactive glass nanoparticles (Sr‐BGNPs) by using the modified Stöber method (Naruphontjirakul, Porter, & Jones, 2018). They investigated the osteogenic response of a murine pre‐osteoblast cell line, MC3T3‐E1, for nanoparticles. Immunohistochemistry staining of Col1a1, osteocalcin (OSC) and osteopontin (OSP) showed that these proteins were expressed in the MC3T3‐E1 cells following 3 weeks of culture. At the same time, the expressions of late osteogenic differentiation markers, OSC and OSP, in the 14% Sr‐BGNPs group were significantly higher than those of 0% Sr‐BGNPs group (Figure 10). These data suggest that Sr‐BGNPs may be beneficial to bone regeneration and used as an inorganic drug for bone regeneration. Therefore, the effect of SNPs on bone regeneration may be applied clinically in the future.

## 4 BIOCOMPATIBILITY

Due to SNPs properties consisting of a large specific surface area, easy surface modification and good biocompatibility facilitate the application and the study for further research of SNPs (Johnston et al., 2000; Ravi Kumar et al., 2004). On the other hand, there are concerns about the inconsistent data reported on SNPs for both in vivo and in vitro studies as well as safety issues (Murugadoss et al., 2017). In addition, a large number of toxicological studies have shown that the toxicity of SNPs is related to their concentration, particle size, surface properties, cell type, and other factors (Kim, Joachim, Choi, & Kim, 2015). Prior to application in nanomedicine, the mechanisms associated with toxicity of SNPs need to be understood in greater detail, and steps need to be taken to minimize health risk.

### 4.1 Toxicological studies—in vitro

SNPs may cause cytotoxic effects in some cell lines through different mechanisms, including apoptosis, autophagy, pyroptosis, oxidative stress, and inflammatory responses. Among these, ROS‐mediated effects are considered to be an important mechanism for SNPs cell damage (Murugadoss et al., 2017). MCM‐41 particles showed active oxides in cells, causing oxidative stress and damage (Fritsch‐Decker, Marquardt, Stoeger, Diabate, & Weiss, 2018; Kusaczuk, Kretowski, Naumowicz, Stypulkowska, & Cechowska‐Pasko, 2018; Petrick, Rosenblat, Paland, & Aviram, 2016). SNPs cause cytotoxicity without increasing ROS levels (Frohlich et al., 2009; Thomassen et al., 2011). In addition, Tao et al. (2008) and Huang et al. (2010) found that SNPs can inhibit cellular respiration, proliferation, and apoptosis. Some studies found that there are other ways in which SNPs can cause cytotoxicity. For instance, SNPs can induce autophagy by damaging the structure of lysosomes, increasing membrane permeability, downregulating the expression of lysosomal proteases and cathepsin B, thus damaging the function of lysosomes (Wang et al., 2017). Although the exact mechanism of autophagy is not clear, SNPs‐induced autophagy is very evident. Recently, SNPs of different sizes were tested for their toxicity in different cells. These studies found SNPs can induce the pyroptosis in primary microglia, macrophages J774A.1 and N9 cells (Du et al., 2019). Cell death through the toxicity of SNP remains to be fully illustrated.

SNPs can cause other adverse effects such as genotoxicity, immunotoxicity, and neurotoxicity. Among these, immunity and genotoxicity are the focus of attention (Chen et al., 2018; Yazdimamaghani, Moos, Dobrovolskaia, & Ghandehari, 2019). At high cytotoxic doses, SNPs can induce the formation of micronuclei (Guichard et al., 2016). These toxicities are consistent in different cell lines and with different SNPs (Gonzalez et al., 2010; Maser et al., 2015). Recent studies have found that SNPs can induce genotoxicity in human tumor cell lines (lung, kidney, skin, and gastrointestinal system), which is related to the induction of oxidative stress (Duan et al., 2013; Guichard et al., 2015; Tarantini et al., 2015). The amplitude of this effect is negatively correlated with the size of the NPs. However, in a study by Guichard et al. (2016), Py‐SNPs (20 nm) induced a significant increase in DNA strand breakage at 100 μg/cm^2^ (24 hr) and c‐SNPs (15 nm) showed a similar effect at 50 or 100 μg/cm^2^. However, none of these SNPs raised ROS levels so the mechanism of the DNA damage by SNPs needs further research (Magdolenova et al., 2014).

The toxic effects of SNPs on the immune system have been inadequately addressed. The properties of SNPs on the cellular uptake by immune cells and the subsequent immune responses need further research. After exposure to NPs, macrophages, dendritic cells, and T‐lymphocytes produce biological responses mediated by inflammatory signaling pathways, ROS, and so on (Chen et al., 2018). Di Cristo et al. (2016) found that Py‐SNPs (~14 nm) induced an increase in tumor necrosis factor‐α, interleukin (IL)‐6 and IL‐1β in RAW.264.7 macrophages. Other results show that with a decrease in particle size, the cytotoxicity of SNPs to Langerhans cells becomes stronger (Nabeshi et al., 2010). It has been found that negatively charged SNPs exert the strongest immunotoxicity in vivo for lymphocytes. They inhibit the proliferation of lymphocytes, inhibit the killing activity of NK cells, and reduce the production of pro‐inflammatory cytokines and nitric oxide (Kim et al., 2014).

### 4.2 Toxicological studies—in vivo

In vivo studies usually involve toxicity studies using animals in which SNPs are administered primarily by inhalation, orally, topically and intravenously. SNPs may be distributed to different parts of the body depending on the route of exposure such as lung, liver, spleen, skin, kidney, and brain tissue (He et al., 2008; Huang et al., 2011; Lu, Liong, Li, Zink, & Tamanoi, 2010). At the same time, NPs have two pathways of excretion from the body. One way is through the kidneys, being filtered out by the urine and the other is through the liver by secreting bile through the feces (Lee et al., 2014).

Some studies showed that SNPs could accumulate in the liver and spleen (He et al., 2008; Linden, 2018; Shao et al., 2017). Liu et al. (2011), using fluorescence and ultrastructural localization techniques, observed that a single intravenous injection of SNPs was distributed to the liver and spleen of animals. The high‐dose (1,280 mg/kg) showed significant degeneration, lymphocytic infiltration, and necrosis. Intraperitoneal injections showed an increase in liver and spleen weight, spleen lymphocyte proliferation as determined by cellular markers (CD3+, CD45+, CD4+, and CD8+), macrophage infiltration, spleen red pulp expansion, and white pulp atrophy (Lee, Kim, Lee, et al., 2013). The smaller the size of the NPs that enters the body through oral or intravenous injection, the easier it is to pass through the kidneys and then excreted in the urine. During this process, SNPs may cause kidney damage, and a significant increase in creatinine levels in the blood, tubular atrophy, necrosis, and renal fibrosis (X. Chen, Zhouhua, et al., 2015; Hao, Li, & Tang, 2014; Li et al., 2015; Shamsi, Ahmed, & Bano, 2017). In other studies, the size of the NPs directly affects the extent of damage to the lung tissue (Handa et al., 2017). Huang et al. (2011) found that PEG‐modified MSNs accumulated in lung tissue, affecting blood circulation time, and up‐regulating the levels of alveolar macrophages. Another study showed that propylamine‐modified MSNs causes lung inflammation, an increase in neutrophils, and may even lead to macrophage cell death (van Rijt et al., 2016).

These results indicated the potential toxicity of SNPs, while other studies have failed to find toxic effects of NPs administration. For example, no toxicity was observed in chronic oral intake and skin exposure studies (Murugadoss et al., 2017). Yun et al. (2015) found that SNPs (12 nm) did not cause abnormal changes in blood biochemical and hematological parameters for acute and subchronic exposed rats. Several studies have also found that different types of SNPs are distributed to different organs but no abnormal symptoms were observed (Heidegger et al., 2016; Huang et al., 2011; Lee et al., 2010). Ryu et al. (2014) exposed rats to l‐arginine coated SNPs (20 nm) for 90 days (6 hr exposure/day) through skin contact at different doses, but no changes in the skin were found. The results were supported by Shim et al. (2014) who performed toxicological studies of effects on the blood–brain barrier at different times after exposing SNPs via oral and dermal. After 28 and 90 days of oral administration of SNPs and after 90 days of percutaneous administration, no significant brain deposition and toxic effects were observed.

To confirm the unfavorable effects of SNPs, more comprehensive and systematic research needs to be conducted. The role of the physicochemical properties of SNPs in vitro and in vivo also needs further studies. The long‐term toxicity and the effect of continuous exposure to health, especially the co‐exposure problems of SNPs and chemical composition need to be addressed. It is also necessary to establish the nanotoxicology related standard about toxicity detection method and practical application. Investigating and producing biodegradable nanoparticles materials is another way to reduce the potential toxicity (Li & Liu, 2017; Su & Kang, 2020).

## 5 CONCLUSION

Nanomedicine has developed rapidly as a consequence of progresses of science and technologies in multiple and varied research approaches. Due to the large specific surface area, easy synthesis and amplification, easy surface modification, and good biocompatibility, SNPs have been a research hotspot in the field of nanomaterials and nanomedicine. In the field of drug delivery, MSNs utilize their own mesoporous structure and porosity advantages to achieve drug delivery possibilities by adjusting the pore size and modifying the surface. Moreover, compared to conventional SNPs, the more advanced SNPs that are now being developed have the advantage of versatility for simultaneous bioimaging and drug delivery. The use of bio‐imaging to monitor the drug delivery in real time can improve medical treatments. In addition, SNPs have relevant applications to several research fields such as PTT, PDT, antibacterial infection, wound healing, and bone regeneration.

While SNPs are widely used, their biosafety issues are of concerned. Both in vitro and in vivo studies showed that SNPs may cause cytotoxicity, hepatosplenic toxicity, genotoxicity, and immunotoxicity. However, in many toxicity studies, the results are inconsistent and the direction of the research is not clear cut. Given the use of SNPs in medical applications, we need to document the biocompatibility and toxicokinetic data in greater detail. Therefore, in order to succeed in the clinical application of SNPs more research is required to develop and standardize the procedures, more comprehensive and detailed research support is needed, and precisely defined the regulatory requirements. At the same time, it is expected that the application of SNPs in nanomedicine will bring more health benefits to human beings in the future.

## CONFLICT OF INTEREST

The authors have declared no conflicts of interest for this article.

## AUTHOR CONTRIBUTIONS

**Ziyuan Li:** Data curation; methodology; writing‐original draft. **Yingwen Mu:** Data curation; writing‐original draft. **Cheng Peng:** Funding acquisition; methodology; writing‐original draft; writing‐review and editing. **Martin Lavin:** Formal analysis; methodology; writing‐review and editing. **Hua Shao:** Data curation; funding acquisition; methodology; writing‐review and editing. **Zhongjun Du:** Data curation; funding acquisition; methodology; resources; writing‐original draft; writing‐review and editing.

## RELATED WIREs ARTICLE

Mesoporous silica nanoparticles for tissue‐engineering applications
